# Are the Australian Dietary Guidelines Fit for Purpose for Culturally Diverse Populations? A Qualitative Study

**DOI:** 10.1002/hpja.70152

**Published:** 2026-01-14

**Authors:** Hyatt Narsh, Danielle Gallegos

**Affiliations:** ^1^ School of Public Health and Social Work, Queensland University of Technology Brisbane Australia; ^2^ Ethnic Communities Council of Queensland Brisbane Australia; ^3^ Centre for Childhood Nutrition Research, Faculty of Health Queensland University of Technology Brisbane Australia; ^4^ School of Exercise and Nutrition Sciences, Queensland University of Technology Brisbane Australia

**Keywords:** cultural tailoring, culturally and linguistically diverse, food‐based dietary guidelines, health equity, nutrition

## Abstract

**Issue Addressed:**

Migration is globally recognised as a determinant of health. The transition for culturally diverse communities towards adopting diets that align with dietary recommendations of their host country is not without complexities. With over one‐quarter of Australia's population being born overseas, this research explores whether the current Australian food‐based dietary guidelines and its communications are relevant and fit for purpose for culturally diverse communities in Australia.

**Methods:**

An exploratory qualitative descriptive study, informed by the Health Equity Framework, was conducted using semi‐structured individual interviews with ‘experts’ in the field of culturally diverse communities, health equity and/or dietary guideline development and analysed using abductive thematic analysis.

**Results:**

Eight interviews were conducted, and three themes were constructed: (1) Bias as a lack of consultation and meaningful data is used in guideline development; (2) Adaptation is possible, but nobody is taking responsibility; and (3) The way forward is through co‐design.

**Conclusion:**

There are currently numerous structural inequities that prevent the dietary guidelines and their communication being fit for purpose for culturally and linguistically diverse Australians. To meet the needs of a culturally diverse population, a health equity approach to food‐based dietary guideline development and adaptation needs to be actioned.

**So What?:**

Whilst there is an understanding that dietary guidelines take a whole‐of‐population approach, review of the guidelines provides an opportunity for federal and state governments to share responsibility to facilitate the development of additional resources for research on diverse dietary patterns, and adaptations of food‐based dietary guideline communications. Genuine collaboration with culturally diverse communities is essential to promote an equitable approach to health that benefits all Australians.

## 1 Introduction

Globally, food‐based dietary guidelines (FBDG) form the basis of national nutrition, health and agricultural national policies that aim to foster healthy eating habits and prevent malnutrition and non‐communicable diseases [1, 2]. Based on rigorous evidence‐based reviews on the impact of diet on health by experts where bias and certainty are assessed, FBDG are government‐led policies that formulate population‐level messaging, providing practical guidance and support with nutritional intake to influence health outcomes [3, 4, 5]. They are the basis of food‐based interventions across sectors, including schools, aged care, agriculture and health services [2]. Therefore, FBDG need to take into consideration the dietary patterns of all population groups, and messaging needs to resonate and be practical for diverse communities. High‐income countries, such as Australia, Canada, United States and New Zealand, typically receive large influxes of migrants, refugees, and asylum seekers contributing to a culturally and linguistically diverse (CaLD) population [6]. Consequently, a ‘one‐size fits all’ approach to FBDG may not be adequate to address these diverse population needs. In Australia, the recent census indicated that over one‐quarter (27.6%) of the population were born overseas, and of these, nearly three‐quarters (71.8%) spoke a language other than English at home [7].

Migration is globally recognised as a determinant of health [8, 9]. The impact of migration can be positive and negative, and is typically poorly understood due to the complexity and diversity of migration journeys, including changes in economic, cultural and social factors [10]. People belonging to cultures and languages that differ markedly from what is considered mainstream (e.g., in Australia this would be anybody who does not identify as white, Anglo‐Celtic and English‐speaking) can have unique needs related to addressing the social determinants of health, including accessing adequate incomes, employment, housing and health services. These unique needs challenge the implementation of population‐wide health strategies. But when an equity lens is used alongside the implementation of evidence‐based policy, it ensures that policies and interventions provide opportunities for all people to attain their highest level of health, regardless of social status or cultural background [11].

Diet‐related behaviour change is complex as it involves the intersection of the social and commercial determinants of health and requires modifications to food that is strongly linked to cultural identity and social practices [12]. The process of migration can impact dietary patterns and food consumption [13]. Termed dietary acculturation, this refers to the cultural, behavioural and psychological changes that take place as migrating communities are exposed to and adapt (or not) to different cultural food environments and habits [14, 15]. Dietary acculturation is not linear and can be variable, ranging from complete assimilation with or the complete rejection of the host culture's food and dietary patterns [16]. Rarely are the ends of the spectrum fully realised but rather cultural foodways are interwoven with adopted practices dependent on context and practicalities [17]. CaLD communities are then faced with trying to access information about how to integrate familiar and unfamiliar foods into their dietary patterns to meet health recommendations—and this is complex. This is further exacerbated by gaps in policy level interventions such as the dietary guidelines that further increase barriers to maintaining traditional eating patterns and supporting the selection of healthy food choices that may be perceived as being outside the norm [13, 18, 19, 20, 21].

Migrating communities have rich culinary traditions that reflect cultural identity and histories. The rituals around the preparation, consumption and sharing of foods hold strong value within culturally diverse communities as they are passed down through generations to preserve this identity [22]. They are deeply intertwined with social customs, and consequently, the attempt to transition into new ways of eating and ideas of health can erode these cultural practices, which have broader social, psychological and health implications [23, 24, 25]. Supporting cultural food practices may lead to positive broader health impacts. Many cultural groups want to maintain their traditional dietary habits but see discrepancies with what is being promoted as ‘healthy’ in their host country. An example can be seen within the Western African community in Australia with the use of palm oil. Palm oil has cultural significance within the community; in addition to its psychosocial benefits, it is also high in vitamin A, which is heavily promoted in West Africa to minimise the risk of deficiency. Upon arrival to Australia, these communities are told to reduce consumption due to the high saturated fat content and the ethical considerations in its production, leading to confusion and disruption of cultural identity [26, 27]. Unfamiliar food environments, in addition to other socioeconomic changes upon migration, can disrupt identity, social connections and hinder cultural food consumption and practices, leading to physical, mental and psychosocial health issues, as well as food insecurity [28]. Therefore, it is imperative to support holistic health approaches by empowering individuals and communities to exercise agency around food choices, which requires culturally relevant messaging and policy [29]. Most FBDGs and their communication developed in countries with histories of colonisation and migration typically fail to reflect the dietary patterns and practices relevant to the diverse communities who now reside in those countries. This is because they are typically based on the most common dietary patterns collected by national nutritional surveys, which tend to have underrepresentation of culturally diverse groups and therefore exclude varied eating patterns [30, 31].

The public health and nutrition communities have largely addressed these challenges through the reliance on the development of interventions that are education‐based and predicated on messaging designed for the whole population to prevent disease [32, 33]. Notwithstanding that a focus on a health education only approach is limited in the general context of behaviour change (see e.g., *Experiences and perspectives of integrating nutrition education into an exercise program for people with chronic medical conditions* [34]) education materials for CaLD communities are often only translated into another language without being adapted to cultural context [35, 36]. Translation plays a crucial role in global communication, but it is not enough as it misses the nuances embedded in culture and within this context, this includes the importance of culturally specific foods and their connection to heritage and holistic health in CaLD communities [37, 38]. Additionally, this translation only approach ignores communities who may be illiterate in their own language, adding additional barriers to meeting health needs [39].

With a predominant education focus, little attention has been paid to the overriding policy documents in the form of FBDGs and how these could be more foundational to ensuring a more equitable food and nutrition support [33]. Development of FBDGs and their communication that fail to consider culturally diverse groups could be at risk of perpetuating inequities in health and are potentially an instrument of structural violence [40]. Structural violence is a complex concept, defined by embedded social, political or economic arrangements within systems that put individuals or populations in harm's way [41]. Such a system is designed to perpetuate the inequitable distribution of privileges in society, disadvantaging some at the expense of others [38], leading to those who are disadvantaged being held responsible for their own poor outcomes rather than addressing the upstream causes [42]. Studies have identified the influence of structural violence impacting CaLD communities within diabetes prevention, food and nutrition security, and responses to Ebola, COVID‐19 and AIDS/HIV [41, 43, 44, 45, 46, 47].

Given the important role of FBDG as a policy document, the aim of this study was to explore if the current Australian Dietary Guidelines (ADGs) and its communication as the Australian Guide to Healthy Eating (AGTHE) are relevant and fit for purpose for CaLD communities living in Australia.

## 2 Methods

This research uses an exploratory qualitative descriptive design, informed by an overarching Health Equity Framework (HEF) [48]. Qualitative description as a methodology is a general interpretive approach that is typically used in health to gain insights into or describe phenomena that are poorly understood [49].

### 2.1 Theoretical Framework

HEFs are based on the understanding that health outcomes are shaped through the dynamic interactions between people and their environment [48, 50, 51]. This includes social, cultural and economic structures and reflects the impact of changes over time. The frameworks recognise that resources and opportunities are not equally distributed amongst the population, and this can manifest as structural violence. These biases may stem from racism, sexism, classism, homophobia, transphobia and ableism, which, when left neglected, can lead to inequities in health outcomes. For this research, the HEF [48] was used as a pragmatic underlying lens, guiding the investigation, informing the questions and analysis of data. It enabled an examination of how societal structures within health may have impacted the access and development of equitable population‐level dietary guidelines and their results in positive health outcomes. The HEF intentionally places population health outcomes in the centre of the model with four spheres of influence: (1) Relationships and Networks, (2) Individual Factors, (3) Systems of Power and (4) Physiological Pathways [48]. Targeting these influences encourages public health practices to go beyond the traditional focus on individual onus of responsibility and addressing the gaps in external structural influences [51]. All four spheres were considered throughout the research development and analysis, with a primary focus on the impact of ‘systems of power’ and its influence on the other three domains and health outcomes overall within the context of migration, dietary patterns and nutrition.

For this research the term CaLD will be used to describe migrants, refugees, asylum seekers and immigrants from culturally diverse communities. The term is commonly used in Australia, but is an umbrella term that conflates 250 cultural and language groups [7]. This term does not include First Nations peoples who are the original inhabitants of the lands now called Australia and who need to be considered separately but who are also experiencing disruption of traditional foodways and structural violence.

### 2.2 Recruitment

Purposive sampling was used to identify potential participants who were considered ‘experts’ in the adaptation or use of dietary guidelines with CaLD populations. Expert status was determined through authorship of papers on any topic related to diet, foods, migration and cultural diversity or via participation in FBDG policy development. Personal networks and snowballing were also used. The aim was to recruit up to 10 participants.

Eligibility included:

- Experience with the adaptation and development of culturally tailored dietary guidelines for CaLD communities.
- Current or prior member of committees who have developed or implemented national dietary guidelines.
- Responsibility for disseminating guidelines at a state or local level.
- Actively teaching or researching food‐based guidelines in a tertiary education setting.

### 2.3 Interviews

Prior to conducting the interviews, a review of the literature and a desktop audit of FBDG development documents from both high‐ and low‐income countries, including Canada, United States of America, New Zealand, United Kingdom, Brazil, Ethiopia, Sri Lanka and Lebanon was conducted (unpublished). This document analysis informed the development of questions for the interviews and provided a nuanced understanding of how cultural practices are managed in different country contexts.

A semi‐structured interview guide was developed based on the findings of a preliminary scoping review using the HEF (described above) as a guide. Semi‐structured interviews were conducted and recorded via *Zoom* by the researcher H.N., informed consent was obtained in writing prior to the commencement of each interview. Recorded interviews were transcribed verbatim through Office Intelligence Services (Version 16.76.1 Microsoft 365), checked for accuracy and corrected based on the audio recording. Transcripts were de‐identified as indicated on participant consent forms.

### 2.4 Ethics

Ethics approval was given by the Queensland University of Technology HREC (#6696). All participants provided written consent prior to or at the interview.

### 2.5 Analysis

The transcribed interviews were analysed using abductive thematic analysis, which combined both inductive and deductive approaches [52, 53, 54]. This method enabled researchers to apply Braun and Clarke's [55] open thematic analysis to identify patterns from the data whilst simultaneously incorporating theory to refine and develop themes. Initially, two interviews were open coded independently by both authors, who then refined the codes through discussion. One author proceeded to code the remaining interviews, after which the authors collaboratively discussed and categorised the codes into overarching themes.

In keeping with qualitative analysis, a reflexivity statement is provided. Both authors identify as cis‐female and are dietetically trained with public health expertise. Narsh identifies as a first generation Syrian Australian, born to Syrian parents who migrated in the 80s. She has worked with CaLD communities for 5 years. Gallegos is white, identifies culturally as Australian with no history of migration. She has worked with CaLD communities for over 30 years. Their experiences and backgrounds led to the development of the research question and their lived and learned expertise contributed to the interpretation and construction of the themes.

## 3 Results

Fifteen potential interviewees who met the criteria were approached by either author to take part. Of those contacted, eight participants were interviewed, with the other seven declining due to conflicts of interest (this included those on current committees or working at the federal government level on nutrition policy) or time limitations. Of the eight interviewed, four had direct experience with adaptation and dissemination of FBDG for CaLD communities at a local or state level, and the remaining four were actively in university teaching or research roles related to FBDG. Through analysis, three themes were constructed: (1) Bias as a lack of consultation and meaningful data to inform guideline development; (2) Adaptation is possible, but nobody is taking responsibility; and (3) The way forward is through co‐design. These three themes were identified in both the context of the FBDG and their communication (i.e., the AGTHE).

### 3.1 Bias as a Lack of Consultation and Meaningful Data to Inform Guideline Development

Participants described underlying bias in the development of the Australian FBDG and the AGTHE that effectively excluded CaLD communities. This bias was demonstrated through lack of authentic consultation with CaLD communities, the failure to incorporate evidence due to primary lack of research and data into CaLD dietary habits within the Australian context, and failure to work with communities on FBDG messaging to ensure equitable access to information.

#### 3.1.1 Lack of Genuine Consultation

Participants commented on how CaLD communities were neglected in the process of developing FBDG, particularly with consultation described as tokenistic or non‐existent for both established and less well‐established communities. The lack of inclusion of people from culturally, linguistically and socio‐economically diverse backgrounds results in eating patterns, food grouping and choices of these communities not being considered or represented in the development of AGTHE and the Australian FBDG. This lack of consultation with a significant proportion of the population reduces community ownership, in turn contributing to limited dissemination and use of guidelines, as they are unrelatable and unrealistic.

So, you've got participation, which can be tokenistic, which is often, in my opinion, what the government does … and they'll say they consulted the community because they've gone and done that. But you know you'll never have these culturally diverse groups engaging in those consultation processes. So that's not true participation, whereas true participation is giving community some sense of ownership over the project so that by the end of it, they feel like they have power in decision making and that they because of that sense of ownership, they'll go to their communities, and they'll disseminate it. (P1)

#### 3.1.2 Lack of Meaningful Robust Research and Data

The lack of high‐quality research that encompasses meaningful robust data from CaLD communities was highlighted as another area where bias is prominent. Participants highlighted the lack of data on diverse eating patterns in Australia and how dietary habits of some CaLD communities can adapt and change upon moving to Australia. The development of the FBDG is based on the review of available evidence that is assessed based on the quality of research and the strength of evidence. Data on dietary patterns, nutrition issues and foods consumed by CaLD communities in Australia have not previously been included as they are typically based on studies with small samples, cross‐sectional studies, and may be of poor evidential quality. It was highlighted that best practice policies are founded on evidence, and if the evidence is not capturing the current population's needs, CaLD communities will continue to be unaccounted for in the development of the FBDG and the AGTHE.

…the surveys and the actual data we're using are never representative of these groups. And increasingly, I think we need to recognise and understand that the actual way we do research is flawed and doesn't include diverse groups nearly as much. (P1)

… there's not much there to help clinicians to use the guidelines for specific culture group and there's not enough information about the diet of specific culture groups even to adapt it… this is what the science says, but it doesn't really correlate with what really people eat now… there's a massive gap in what is best practice and the reality of Australian's life. (P2)

### 3.2 Adaptation Is Possible, but Nobody Is Taking Responsibility

There have been attempts at developing communications about ‘healthy’ eating for CaLD communities, but this was seen to have been done in a ‘tokenistic’ way. Many of the participants used the example of the AGTHE being directly translated to a limited number of languages, without adaptations or tailoring to specific community foods and needs. Participants described consultations as ‘ticking a box’, with communities defined under general descriptors (e.g., Asian or African or Middle Eastern) making invisible diet pattern diversity. A key message was that again, attempts to collaborate with CaLD communities to adapt messages had failed to adequately and genuinely engage them.

If the evidence still says that we should have 5 serves of vegetables every day, we need some materials that demonstrate that those vegetables don't have to be the vegetables that are typically consumed by, you know, white middle class Australians. (P6)

The dietary guidelines have been developed in principle to apply to all Australians. That's what they say but that doesn't mean that they're very useful or applicable to all Australians.

Yeah. I mean, it's an equity or, uh, ethical issue, really. I think that, if this information is made available for majority, perhaps I'm not even sure that the food patterns are most food patterns anymore that they currently represent. But, you know, every group of society needs to have equal access to that information, so it's just an equity [issue]. (P5)

Participants made the point that one of the reasons why the AGTHE has not been tailored and adapted to meet the needs of CaLD populations is there are no lines of responsibility and/or no single agency is responsible for funding to undertake this task. Participants agreed that tailoring the guidelines requires collaboration between federal institutions, who develop the guidelines, and state and grassroots organisations who are responsible for dissemination and that responsibility should not fall on one organisation. Participants acknowledged that adaptations require economic and labour‐intensive resources as well as knowledge to execute appropriately whilst also ensuring that messaging remains aligned with the evidence. A division of responsibilities is required across federal, state and community organisations through the development, consultation and reviewing process for FBDG and well as during tailoring and creation of adapted resources and dissemination of messaging.

The government needs to lead it and provide the funding for it, but I think it is the work of [all] communities. So, you go back to the principle of public health. We have to work with the system first and then stakeholder and communities. But everyone, everyone now needs to play a role … communities … should be invited, should have a say, you know and through multiple channels. The government, could, lead it and needs to make sure that they have the funding and the resources. But everyone, everyone now needs to play a role. (P3)

### 3.3 The Way Forward Is Co‐Design

A co‐design approach was highlighted by all participants as the best way to engage communities to be included in the development and adaption of FBDG messaging and resources. This theme acknowledges that previous approaches have failed to equitably include communities and that a shift to a co‐design would be more resource heavy, but would create equitable changes to guidelines and their communication. A co‐design approach would engage communities, building trust and rapport in contributing to FBDG that may be more likely to be used by CaLD communities. As mentioned, this method breaks the history of tokenistic outreach and creates opportunity for ownership on the content and dissemination, leading to higher levels of utilisation, greater integration of policy into everyday practice and potentially higher prevalence of diets that are health promoting.

I think a lot of communities are quite open and want to provide that input and support, but value it you know just don't ask for the sake of asking it all the time, you know, but having a clear, transparent process, rewarding them, you know, rewarding them for being part of that process … then in terms of how we can actually work with the community, we can actually ensure that the future guideline is applicable to a diverse range or to you know, all Australian communities. I think that ongoing work and co‐design with them having their genuine interest in that, but understanding and that we need dollars. (P3)

Once you do give people an opportunity to have a say on things and actually listen to them, ask them what healthy means to them and incorporate that into the guidelines and continue that … Co design process that people are talking about, which often means going back to the community a few times. It just takes time, but that's how you do it properly. It's just that people like to cut corners. (P1)

In summary, participants who were involved in working with the FBDG and CaLD communities described the process of FBDG and AGTHE development as failing to be inclusive of diversity. The lack of evidence and genuine engagement with CaLD communities resulted in guidelines and messaging that were not relevant or fit for purpose. This is a missed opportunity to broadly influence dietary patterns towards a more healthful approach for CaLD communities living in Australia.

## 4 Discussion

Interviews with people experienced in the adaptation or use of dietary guidelines with CaLD populations revealed that the current Australian FBDG and their communications as the AGTHE may not be relevant or fit for purpose for many CaLD Australians. The Australian FBDG aims to provide high‐level evidence to formulate guidelines and messaging appropriate for the entire Australian population. However, it fails to consider the unique dietary preferences, traditions and cultural backgrounds of culturally diverse populations in Australia within the research that underpins the guidelines and in consultation and dissemination processes. This lack of genuine representation in policy perpetuates inherent bias and builds on the potential inequities that are already present within the Australian health system [56].

Using the HEF, the findings from these interviews indicate that for CaLD Australians there are missed opportunities that fail to capitalise on: (1) the relationships and networks of CaLD communities to promote health‐enabling choices; (2) the assets of CaLD communities and individuals with respect to their unique attitudes, skills and behaviours; and (3) promoting health equity through the genuine inclusion of CaLD communities in the development, dissemination and implementation of guidelines [48]. This failure has become institutionalised, that is, the inequitable processes and practices are now embedded such that it is invisible to those with power. As such, the development of FBDG and the AGTHE could be seen as a form of structural violence actively hampering the ability of CaLD communities to make health‐enabling choices [57]. This places the onus of responsibility with the individual, and so those who are excluded and marginalised are held accountable for their poor health outcomes rather than the underlying upstream contributors being addressed [42, 58].

The development of FBDG and their interpretation into communication resources is a significant public health endeavour [1]. Such guidelines are considered ‘best practice’, as they are based on research assessed for its quality, with the goal to create positive health outcomes for populations [2]. Within the Australian context, the NHMRC utilises the GRADE (Grading of Recommendations, Assessment, Development, and Evaluations) framework to assess the inclusion of quality research for synthesis [59]. Research is evaluated for inclusion/exclusion across four domains: risk of bias, imprecision, inconsistency and indirectness [60]. With acknowledgement that FBDG are designed to apply to populations, the dietary patterns and cultural nuances around food in CaLD communities are not fully integrated into these population health policies partially due to the lack of available research that would meet the quality benchmark for inclusion. The evidence often fails to keep pace with demographic changes, globalisation and the diversity of eating habits within contemporary societies, leading to a misalignment between reality and guidelines, that are therefore not representative of the population [61, 62, 63]. When compared to other high‐income, high migration countries such as Canada and the United Kingdom, Australia's FBDG and communications incorporate some diversity through foundation diets modelling that included omnivore, lacto‐ovo vegetarian, pasta and rice‐based patterns [64, 65, 66]. This range of diets provides some considerations for diverse eating patterns, but still fails to embrace the range of dietary patterns within the Australian context. The United States Dietary Guideline committee has recently released a scientific report to inform the creation of the 2025–2030 FBDG. This report highlighted the importance of future guidelines to be representative of the population's culturally diverse nutritional needs and preferences, calling for consideration of culturally responsive intervention research to be included to inform the importance of diet flexibility and diverse dietary habits [67]. The combination of minimal available data from Australian CaLD communities on dietary intake and patterns, and the lack of genuine consultation with diverse communities, continues to perpetuate structural health inequalities in FBDG development in the Australian context [31, 40, 68, 69].

Addressing the health of CaLD communities is often labelled challenging due to the complexity in addressing individual community nuances, particularly around food and its connections to cultural identity. Consequently, institutions that drive nutrition policy and public health initiatives fail to understand these implications and their impact on health outcomes. Not engaging CaLD communities can lead to institutionalised biases [40, 70]. The Australian FBDG and AGTHE are examples of how policy that informs not only individual and organisational (hospitals, schools, aged care) practices has failed to embrace cultural diversity and are therefore inequitable. When health policies mask systemic exclusion through a ‘one‐size‐fits‐all’ approach, they fail to reflect the specific and diverse health needs of the population [71]. Disregarding the impact of culture and migration perpetuates the inequities in the health system [72, 73]. These inbuilt inequities result in the responsibility for promoting dietary health to fall on grassroots, community organisations or ‘community heroes’. These groups and individuals are expected to bridge systemic gaps with limited resources through the creation of culturally relevant resources and programmes driven by their commitment to support their communities [74]. This dynamic, which exploits the goodwill of the communities, allows governments and policy development institutions to absolve themselves of responsibility. By claiming that FBDG are flexible and suitable for everyone, the onus is placed back on communities to interpret and adapt these guidelines to meet their needs [75].

A key solution to embedding health equity in FBDG development lies in meaningful collaboration with CaLD communities. Grassroots, co‐design approaches, in which communities are active and resourced participants in designing and adapting policy, ensure that their voices, experiences and cultural practices shape nutrition guidelines and consumer resources. A co‐designed approach encompasses all dimensions relevant to addressing health needs, including the individual and community level, co‐creating a cohesive message free of stereotypes and suitable to cultural needs around food [36, 38, 76, 77, 78]. Examples of CaLD co‐designed/tailored approaches can be where initiatives, such as the *Good Start Program*, *Pasifika Women's Diabetes Wellness Program* and *Living Well Multicultural*, have been created by community for community or have been specifically tailored to address needs around nutrition, physical activity and chronic condition prevention and management [79, 80, 81]. These programme evaluations highlight how designing and tailoring through well‐resourced, community‐led approaches that adequately address community needs, values and beliefs around health can create positive change. Examples like these have consistently shown to enhance positive health outcomes within culturally diverse communities, highlighting lived experiences and being able to ensure cultural sensitivity, ‘authentic’ representation and foster ownership of the work, supports these outcomes as it becomes part of the fabric of a community [82, 83, 84, 85, 86]. These examples show how it is possible to invest in a genuine co‐design method and achieve equitable impacts in health and give the opportunity to reflect on how the strategies utilised could be expanded to a policy level and in the review of the ADG and its communications in future.

## 5 Limitations

One of the primary limitations of the study is the small sample size and lack of participant diversity focussing only on those with predominantly learned rather than lived expertise [87]. Those interviewed were limited to academics and professionals involved in FBDG development as the focus was on the development of policy. Participants with frontline experience, including multicultural health workers, dietitian‐nutritionists and health promotion practitioners, may have additional insights into the practicalities of co‐design with communities. Highlighting their voices and experiences in this paper would have added an additional lens, outside of academia and policy. Understanding the practicality of utilising the FBDG in CaLD nutrition spaces needs further interrogation and research. Interviewing those with professional expertise is only one perspective; further research with members of CaLD communities would provide additional insights. Due to conflicts of interest, we were unable to recruit anybody involved in the development of the ADGs. Additionally, both researchers were dietetically trained and as such likely contributed to the interpretation of the data in a particular way. This study is a first step to start the discussion of equity in nutrition policy within a globalised world.

## 6 Conclusion

Failing to account for cultural diversity and the diet changes that occur on migration and avoiding genuine collaboration with CaLD communities means the FBDG's will continue to reinforce the systemic barriers that limit positive health outcomes. The reliance on community champions to address these gaps further exacerbates disparities, shifting responsibility away from institutions and onto under‐resourced actors. To address these challenges, systemic change is essential. A HEF identifies that genuine collaboration and partnership with CaLD communities, additional research funding focused on CaLD dietary patterns and nutritional status, alongside increased resourcing for co‐design of messaging, are critical pathways to creating inclusive, evidence‐based nutrition policies. By prioritising equity and dismantling structural violence, policymakers can better support the health and wellbeing of all population groups, ensuring that no one is left behind.

## Funding

The authors have nothing to report.

## Ethics Statement

Ethics approval was given by the QUT HREC (#6696).

## Consent

All participants provided written consent prior to or at the interview.

## Conflicts of Interest

The authors declare no conflicts of interest.

## Data Availability

Research data are not shared.

## References

[1] C. Gonzalez Fischer and T. Garnett , Plates, Pyramids, Planet. Developments in National Healthy and Sustainable Dietary Guidelines: A State of Play Assessment (FAO, 2016).

[2] A. Herforth , M. Arimond , C. Álvarez‐Sánchez , J. Coates , K. Christianson , and E. Muehlhoff , “A Global Review of Food‐Based Dietary Guidelines,” Advances in Nutrition 10, no. 4 (2019): 590–605. 31041447 10.1093/advances/nmy130 PMC6628851

[3] S. S. Gropper , “The Role of Nutrition in Chronic Disease,” Nutrients 15, no. 3 (2023): 664. 36771368 10.3390/nu15030664 PMC9921002

[4] R. W. Kimokoti and B. E. Millen , “Nutrition for the Prevention of Chronic Diseases,” Medical Clinics of North America 100, no. 6 (2016): 1185–1198. 27745589 10.1016/j.mcna.2016.06.003

[5] O. Ojo , “Nutrition and Chronic Conditions,” Nutrients 11, no. 2 (2019): 459. 30813286 10.3390/nu11020459 PMC6412662

[6] M. Czaika and H. de Haas , “The Globalization of Migration: Has the World Become More Migratory?,” International Migration Review 48, no. 2 (2014): 283–323.

[7] Australian Bureau of Statistics , Cultural Diversity: Census (ABS, 2021).

[8] H. Castañeda , S. M. Holmes , D. S. Madrigal , M.‐E. D. Young , N. Beyeler , and J. Quesada , “Immigration as a Social Determinant of Health,” Annual Review of Public Health 36, no. 1 (2015): 375–392. 10.1146/annurev-publhealth-032013-182419 25494053

[9] A. A. Davies , A. Basten , and C. Frattini , “Migration: A Social Determinant of the Health of Migrants,” Eurohealth 16, no. 1 (2009): 10–12.

[10] K. Wickramage , J. Vearey , A. B. Zwi , C. Robinson , and M. Knipper , “Migration and Health: A Global Public Health Research Priority,” BMC Public Health 18, no. 1 (2018): 987. 30089475 10.1186/s12889-018-5932-5 PMC6083569

[11] P. Braveman , E. Arkin , T. Orleans , D. Proctor , and A. Plough , What Is Health Equity? And What Difference Does a Definition Make? (Robert Wood Johnson Foundation, 2017).

[12] R. Shepherd and R. Shepherd , “Resistance to Changes in Diet,” Proceedings of the Nutrition Society 61, no. 2 (2002): 267–272. 12133209 10.1079/PNS2002147

[13] G. Holmboe‐Ottesen and M. Wandel , “Changes in Dietary Habits After Migration and Consequences for Health: A Focus on South Asians in Europe,” Food & Nutrition Research (2012): 56–57. 10.3402/fnr.v56i0.18891 PMC3492807 23139649

[14] J. W. Berry , “Acculturation,” in Reference Module in Neuroscience and Biobehavioral Psychology (Elsevier, 2017).

[15] E. A. Delgado‐Romero , D. Mollen , and C. R. Ridley , “Racial and Ethnic Minorities, Counseling of,” in Encyclopedia of Applied Psychology, ed. C. D. Spielberger (Elsevier, 2004), 211–215.

[16] N. F. Rule , C. C. Dring , and T. F. Thornton , “Meals in the Melting‐Pot: Immigration and Dietary Change in Diversifying Cities,” Appetite 168 (2022): 105728. 34606941 10.1016/j.appet.2021.105728

[17] M. Schumann , M. Bug , K. Kajikhina , et al., “The Concept of Acculturation in Epidemiological Research Among Migrant Populations: A Systematic Review,” SSM – Population Health 10 (2020): 100539. 32042888 10.1016/j.ssmph.2020.100539 PMC6997899

[18] J. C. Banna , B. Gilliland , M. Keefe , and D. Zheng , “Cross‐Cultural Comparison of Perspectives on Healthy Eating Among Chinese and American Undergraduate Students,” BMC Public Health 16 (2016): 1–12. 27669822 10.1186/s12889-016-3680-y PMC5037860

[19] S. D. Lee , N. J. Kellow , C. E. Huggins , and T. S. T. Choi , “How and Why Diets Change Post‐Migration: A Qualitative Exploration of Dietary Acculturation Among Recent Chinese Immigrants in Australia,” Nutrients 14, no. 17 (2022): 3573. 36079830 10.3390/nu14173573 PMC9460769

[20] G. Reddy and R. M. van Dam , “Food, Culture, and Identity in Multicultural Societies: Insights From Singapore,” Appetite 149 (2020): 104633. 32084519 10.1016/j.appet.2020.104633

[21] A. A. F. Carioca , B. Gorgulho , J. A. Teixeira , R. M. Fisberg , and D. M. Marchioni , “Dietary Patterns in Internal Migrants in a Continental Country: A Population‐Based Study,” PLoS One 12, no. 10 (2017): e0185882. 29036177 10.1371/journal.pone.0185882 PMC5642885

[22] N. Ishak , M. S. Mohd Zahari , M. S. Shazali , R. Muhammad , and S. Hannita , “Acculturation, Foodways and Malaysian Food Identity,” in Current Issues in Hospitality and Tourism (Taylor & Francis, 2012), 359–363.

[23] Y. Chen , “Food, Race, and Ethnicity,” in The Oxford Handbook of Food History, ed. J. M. Pilcher (Oxford University Press, 2012), 428–443.

[24] N. Ishak , M. S. M. Zahari , S. A. Talib , and H. M. Hanafiah , “The Influence of Biculturalism/Integration Attributes on Ethnic Food Identity Formation,” Journal of Ethnic Foods 6, no. 1 (2019): 21.

[25] S. D. Lee , N. J. Kellow , T. S. T. Choi , and C. E. Huggins , “Assessment of Dietary Acculturation in East Asian Populations: A Scoping Review,” Advances in Nutrition 12, no. 3 (2021): 865–886. 33119743 10.1093/advances/nmaa127 PMC8166541

[26] D. Gallegos , “Palm Oil Tensions,” New Formations 2011, no. 74 (2011): 18–32.

[27] M. D. Pashkevich , C. A. M. Marshall , B. Freeman , et al., “The Socioecological Benefits and Consequences of Oil Palm Cultivation in Its Native Range: The Sustainable Oil Palm in West Africa (SOPWA) Project,” Science of the Total Environment 926 (2024): 171850. 38521255 10.1016/j.scitotenv.2024.171850

[28] V. Sibal , “Food: Identity of Culture and Religion,” Scholarly Research Journal for Interdisciplinary Studies 6 (2018): 10908–10915.

[29] J. V. Varre , M. Dustin , and S. Van Vliet , “Dietary Transformations and Health Implications in Migrant Populations: A Global Perspective,” Frontiers in Nutrition 12 (2025): 1623556. 40901297 10.3389/fnut.2025.1623556 PMC12400961

[30] K. Wingrove , M. Lawrence , C. Russell , and S. McNaughton , “Evidence Use in the Development of the Australian Dietary Guidelines: A Qualitative Study,” Nutrients 13 (2021): 3748. 34836004 10.3390/nu13113748 PMC8620517

[31] S. H. Ali , N. F. Lin , and S. S. Yi , “Challenging Dietary Research Measures, Concepts, and Definitions to Promote Greater Inclusivity of Immigrant Experiences: Considerations and Practical Recommendations,” Journal of the Academy of Nutrition and Dietetics 123, no. 11 (2023): 1533–1540. 37348677 10.1016/j.jand.2023.06.280 PMC10592485

[32] T. Gingell , K. Murray , I. Correa‐Velez , and D. Gallegos , “Determinants of Food Security Among People From Refugee Backgrounds Resettled in High‐Income Countries: A Systematic Review and Thematic Synthesis,” PLoS One 17, no. 6 (2022): e0268830. 35653308 10.1371/journal.pone.0268830 PMC9162305

[33] H. A. Nur , A. T. Atoloye , H. Wengreen , et al., “A Scoping Review and Assessing the Evidence for Nutrition Education Delivery Strategies for Refugees in High‐Income Countries,” Advances in Nutrition 12, no. 6 (2021): 2508–2524. 34245153 10.1093/advances/nmab080 PMC8634542

[34] H. Powter , K. Lambert , and N. Nicholls , “Experiences and Perspectives of Integrating Nutrition Education Into an Exercise Program for People With Chronic Medical Conditions,” Health Promotion Journal of Australia 35, no. 4 (2024): 1098–1115. 38200682 10.1002/hpja.841

[35] S. Redman and C. Paul , “A Review of the Effectiveness of Print Material in Changing Health‐Related Knowledge, Attitudes and Behaviour,” Health Promotion Journal of Australia 7, no. 2 (1997): 91–99.

[36] M. W. Kreuter , S. N. Lukwago , D. C. Bucholtz , E. M. Clark , and V. Sanders‐Thompson , “Achieving Cultural Appropriateness in Health Promotion Programs: Targeted and Tailored Approaches,” Health Education & Behavior 30, no. 2 (2003): 133–146. 12693519 10.1177/1090198102251021

[37] D. Le , “Investigating How Cultural Differences Influence the Translation Process and the Strategies Used by Translators to Bridge Cultural Gaps,” Journal of Translation and Language Studies 5 (2024): 26–36.

[38] M. K. Lapinski , J. G. Oetzel , S. Park , and A. J. Williamson , “Cultural Tailoring and Targeting of Messages: A Systematic Literature Review,” Health Communication 40 (2025): 808–821. 38961665 10.1080/10410236.2024.2369340

[39] M. Pandey , R. G. Maina , J. Amoyaw , et al., “Impacts of English Language Proficiency on Healthcare Access, Use, and Outcomes Among Immigrants: A Qualitative Study,” BMC Health Services Research 21, no. 1 (2021): 741. 34311712 10.1186/s12913-021-06750-4 PMC8314461

[40] C. Zorbas , J. Browne , A. Chung , et al., “National Nutrition Policy in High‐Income Countries: Is Health Equity on the Agenda?,” Nutrition Reviews 79, no. 10 (2021): 1100–1113. 33230539 10.1093/nutrit/nuaa120

[41] P. E. Farmer , B. Nizeye , S. Stulac , and S. Keshavjee , “Structural Violence and Clinical Medicine,” PLoS Medicine 3, no. 10 (2006): e449. 17076568 10.1371/journal.pmed.0030449 PMC1621099

[42] S. Guillot‐Wright , E. Cherryhomes , L. Wang , and M. Overcash , “Systems and Subversion: A Review of Structural Violence and Im/Migrant Health,” Current Opinion in Psychology 47 (2022): 101431. 36001924 10.1016/j.copsyc.2022.101431

[43] A. J. Odhiambo , P. O'Campo , L. R. E. Nelson , L. Forman , and D. Grace , “Structural Violence and the Uncertainty of Viral Undetectability for African, Caribbean and Black People Living With HIV in Canada: An Institutional Ethnography,” International Journal for Equity in Health 22, no. 1 (2023): 33. 36797746 10.1186/s12939-022-01792-4 PMC9935247

[44] S. Hamed , S. Thapar‐Björkert , H. Bradby , and B. M. Ahlberg , “Racism in European Health Care: Structural Violence and Beyond,” Qualitative Health Research 30, no. 11 (2020): 1662–1673. 32546076 10.1177/1049732320931430 PMC7410275

[45] M. Z. Sharif , J. J. García , U. Mitchell , E. D. Dellor , N. J. Bradford , and M. Truong , “Racism and Structural Violence: Interconnected Threats to Health Equity,” Frontiers in Public Health 9 (2022): 676783. 35186857 10.3389/fpubh.2021.676783 PMC8850294

[46] G. Macassa , C. McGrath , M. Rashid , and J. Soares , “Structural Violence and Health‐Related Outcomes in Europe: A Descriptive Systematic Review,” International Journal of Environmental Research and Public Health 18, no. 13 (2021): 6998. 34208879 10.3390/ijerph18136998 PMC8296855

[47] R. Lindberg , M. Hayley , H. Brontë , and F. H. McKay , “An Investigation of Structural Violence in the Lived Experience of Food Insecurity,” Critical Public Health 33, no. 2 (2023): 185–196.

[48] A. Peterson , V. Charles , D. Yeung , and K. Coyle , “The Health Equity Framework: A Science‐ and Justice‐Based Model for Public Health Researchers and Practitioners,” Health Promotion Practice 22, no. 6 (2021): 741–746. 32814445 10.1177/1524839920950730 PMC8564233

[49] H. Kim , J. S. Sefcik , and C. Bradway , “Characteristics of Qualitative Descriptive Studies: A Systematic Review,” Research in Nursing & Health 40, no. 1 (2017): 23–42. 27686751 10.1002/nur.21768 PMC5225027

[50] C. L. Lewis , A. Yan , M. Y. Williams , et al., “Health Equity: A Concept Analysis,” Nursing Outlook 71, no. 5 (2023): 102032. 37683597 10.1016/j.outlook.2023.102032

[51] L. C. Liburd , J. E. Hall , J. J. Mpofu , S. M. Williams , K. Bouye , and A. Penman‐Aguilar , “Addressing Health Equity in Public Health Practice: Frameworks, Promising Strategies, and Measurement Considerations,” Annual Review of Public Health 41 (2020): 417–432. 10.1146/annurev-publhealth-040119-094119 31900101

[52] J. Thompson , “A Guide to Abductive Thematic Analysis,” Qualitative Report 27 (2022): 1410–1421.

[53] M. Naeem , W. Ozuem , K. Howell , and S. Ranfagni , “A Step‐by‐Step Process of Thematic Analysis to Develop a Conceptual Model in Qualitative Research,” International Journal of Qualitative Methods 22 (2023): 16094069231205789.

[54] S. Timmermans and I. Tavory , “Theory Construction in Qualitative Research: From Grounded Theory to Abductive Analysis,” Sociological Theory 30, no. 3 (2012): 167–186.

[55] V. Braun and V. Clarke , “Using Thematic Analysis in Psychology,” Qualitative Research in Psychology 3, no. 2 (2006): 77–101.

[56] S. Willey , K. Desmyth , and M. Truong , “Racism, Healthcare Access and Health Equity for People Seeking Asylum,” Nursing Inquiry 29, no. 1 (2022): e12440. 34312941 10.1111/nin.12440

[57] J. Page‐Reeves , J. Niforatos , S. Mishra , L. Regino , A. Gingrich , and R. Bulten , “Health Disparity and Structural Violence: How Fear Undermines Health Among Immigrants at Risk for Diabetes,” Journal of Health Disparities Research and Practice 6, no. 2 (2013): 30–47. 24052924 PMC3775498

[58] B. L. Needham , T. Ali , K. L. Allgood , A. Ro , J. L. Hirschtick , and N. L. Fleischer , “Institutional Racism and Health: A Framework for Conceptualization, Measurement, and Analysis,” Journal of Racial and Ethnic Health Disparities 10, no. 4 (2023): 1997–2019. 35994173 10.1007/s40615-022-01381-9 PMC9395863

[59] NHMRC , “Guidelines for Guidelines: Assessing Certainty of Evidence 2019” [updated September 6, 2019], https://nhmrc.gov.au/guidelinesforguidelines/develop/assessing‐certainty‐evidence.

[60] H. Balshem , M. Helfand , H. J. Schünemann , et al., “GRADE Guidelines: 3. Rating the Quality of Evidence,” Journal of Clinical Epidemiology 64, no. 4 (2011): 401–406. 21208779 10.1016/j.jclinepi.2010.07.015

[61] K. H. Onarheim , K. Wickramage , D. Ingleby , S. Subramani , and I. Miljeteig , “Adopting an Ethical Approach to Migration Health Policy, Practice and Research,” BMJ Global Health 6, no. 7 (2021): e006425. 10.1136/bmjgh-2021-006425 PMC8319989 34321236

[62] C. Pérez‐Rodrigo and M. Tseng , “Are Dietary Guidelines Sensible to Consumers?,” Public Health Nutrition 16, no. 5 (2013): 761–762. 23570876 10.1017/S136898001300089X PMC10273245

[63] A. M. N. Renzaho , “The Lack of Race and Ethnicity Data in Australia—A Threat to Achieving Health Equity,” International Journal of Environmental Research and Public Health 20, no. 8 (2023): 5530. 37107811 10.3390/ijerph20085530 PMC10138746

[64] NHMRC , Australian Dietary Guidelines (National Health and Medical Research Council, 2013).

[65] Health Canada , Canada's Dietary Guidelines, ed. Health Canada (Health Canada, 2019).

[66] Public Health England , Eatwell Guide, ed. NHS (NHS, 2018).

[67] Dietary Guidelines Advisory Committee , Scientific Report of the 2025 Dietary Guidelines Advisory Committee: Advisory Report to the Secretary of Health and Human Services and Secretary of Agriculture (U.S. Department of Health and Human Services, 2024).

[68] R. Tiffin and M. Salois , “Inequalities in Diet and Nutrition,” Proceedings of the Nutrition Society 71, no. 1 (2012): 105–111. 22054306 10.1017/S0029665111003284

[69] A. Renzaho , M. Polonsky , D. Mellor , and S. Cyril , “Addressing Migration‐Related Social and Health Inequalities in Australia: Call for Research Funding Priorities to Recognise the Needs of Migrant Populations,” Australian Health Review 40, no. 1 (2016): 3–10. 26164042 10.1071/AH14132

[70] B. H. Kara and N. G. Khawaja , “Wellbeing Programs for Culturally and Linguistically Diverse Population in Australia: Barriers and Improvements,” Australian Psychologist 59, no. 3 (2024): 200–211.

[71] M. Fisher , P. Harris , T. Freeman , et al., “Implementing Universal and Targeted Policies for Health Equity: Lessons From Australia,” International Journal of Health Policy and Management 11, no. 10 (2022): 2308–2318. 34821141 10.34172/ijhpm.2021.157 PMC9808267

[72] M. E. Tinetti , M. Hladek , and D. Ejem , “One Size Fits All—An Underappreciated Health Inequity,” JAMA Internal Medicine 184, no. 1 (2024): 7–8. 37983054 10.1001/jamainternmed.2023.6035

[73] C. P. Duggan , A. Kurpad , F. C. Stanford , B. Sunguya , and J. C. Wells , “Race, Ethnicity, and Racism in the Nutrition Literature: An Update for 2020,” American Journal of Clinical Nutrition 112, no. 6 (2020): 1409–1414. 33274358 10.1093/ajcn/nqaa341 PMC7727473

[74] A. Wennerstrom and D. O. Smith , “Labour Exploitation Among Community Health Workers,” Lancet Global Health 11, no. 10 (2023): e1484–e1485. 37734782 10.1016/S2214-109X(23)00409-6

[75] K. Holden , T. Akintobi , J. Hopkins , et al., “Community Engaged Leadership to Advance Health Equity and Build Healthier Communities,” Social Sciences (Basel, Switzerland) 5, no. 1 (2016): 2. 27713839 10.3390/socsci5010002 PMC5048675

[76] M. Barrera, Jr. , F. G. Castro , L. A. Strycker , and D. J. Toobert , “Cultural Adaptations of Behavioral Health Interventions: A Progress Report,” Journal of Consulting and Clinical Psychology 81, no. 2 (2013): 196–205. 22289132 10.1037/a0027085 PMC3965302

[77] M. W. Kreuter and C. S. Skinner , “Tailoring: What's in a Name?,” Health Education Research 15, no. 1 (2000): 1–4. 10788196 10.1093/her/15.1.1

[78] C. Mead , R. Tindall , and K. Charlton , “Evaluation of a Nutrition Education Resource for Refugees and Newly Arrived Migrants to Australia,” Health Promotion Journal of Australia 34, no. 2 (2023): 536–543. 35642434 10.1002/hpja.625

[79] H. Akbar , M. Contor , W. Niumata , D. Anderson , and D. Gallegos , “Effectiveness of the Pasifika Women's Diabetes Wellness Program (PWDWP): Protocol for a Pilot Intervention and Feasibility Randomized Controlled Trial,” JMIR Research Protocols 13 (2024): e55435. 38286130 10.2196/55435 PMC10964139

[80] S. Mihrshahi , L. Vaughan , N. Fa'avale , S. De Silva Weliange , I. Manu‐Sione , and L. Schubert , “Evaluation of the Good Start Program: A Healthy Eating and Physical Activity Intervention for Maori and Pacific Islander Children Living in Queensland, Australia,” BMC Public Health 17, no. 1 (2017): 77. 28086843 10.1186/s12889-016-3977-x PMC5237210

[81] D. Gallegos , H. Do , Q. G. To , B. Vo , J. Goris , and H. Alraman , “The Effectiveness of Living Well Multicultural‐Lifestyle Management Program Among Ethnic Populations in Queensland, Australia,” Health Promotion Journal of Australia 32, no. 1 (2021): 84–95. 32053254 10.1002/hpja.329

[82] A. Chauhan , J. Leefe , É. N. Shé , and R. Harrison , “Optimising Co‐Design With Ethnic Minority Consumers,” International Journal for Equity in Health 20, no. 1 (2021): 240. 34736455 10.1186/s12939-021-01579-z PMC8567634

[83] A. P. Moore , S. H. Stanton‐Fay , C. A. Rivas , S. Harding , and L. M. Goff , “Co‐Design of a Culturally‐Tailored Diet & Lifestyle Intervention for Diabetes Management in the UK African‐Caribbean Community,” Proceedings of the Nutrition Society 76, no. OCE4 (2017): E163.

[84] R. Harrison , M. Walton , U. Chitkara , et al., “Beyond Translation: Engaging With Culturally and Linguistically Diverse Consumers,” Health Expectations 23, no. 1 (2020): 159–168. 31625264 10.1111/hex.12984 PMC6978859

[85] A. MacFarlane , S. Huschke , M. J. Marques , et al., “Normalising Participatory Health Research Approaches in the WHO European Region for Refugee and Migrant Health: A Paradigm Shift,” Lancet Regional Health. Europe 41 (2024): 100837. 39119099 10.1016/j.lanepe.2024.100837 PMC11306213

[86] H. Akbar , I. Chow , W. Nuimata , A. W. Te Kani , A. V. Langan , and D. Gallegos , eds., “Development of a Pasifika Women's Diabetes Wellness Program: From the Perspective of Māori and Pasifika Women Living in Queensland,” in *Queensland Women's Heath Forum* (The Australian Health Research Alliance (AHRA) WHRTN, 2021).

[87] D. Gallegos and M. M. Chilton , “Re‐Evaluating Expertise: Principles for Food and Nutrition Security Research, Advocacy and Solutions in High‐Income Countries,” International Journal of Environmental Research and Public Health 16, no. 4 (2019): 561. 30769953 10.3390/ijerph16040561 PMC6406792

